# The Effects of Polyphenol, Tannic Acid, or Tannic Acid in Combination with Pamidronate on Human Osteoblast Cell Line Metabolism

**DOI:** 10.3390/molecules27020451

**Published:** 2022-01-11

**Authors:** Hermizi Hapidin, Nor Munira Hashim, Mohamad Zahid Kasiram, Hasmah Abdullah

**Affiliations:** Biomedicine Programme, School of Health Sciences, Health Campus, Universiti Sains Malaysia, Kubang Kerian 16150, Malaysia; normunira94@gmail.com (N.M.H.); zahidkasiram@gmail.com (M.Z.K.); hasmahab@usm.my (H.A.)

**Keywords:** bone sialoprotein, osteoblast, osterix, pamidronate, synergism, tannic acid

## Abstract

Background: This study investigates the effect of tannic acid (TA) combined with pamidronate (PAM) on a human osteoblast cell line. Methods: EC_50_ for TA, PAM, and different combination ratios of TA and PAM (25:75, 50:50, 75:25) were measured by 3-(4,5-dimethyl-2-thiazolyl)-2,5-diphenyl-2H-tetrazolium bromide (MTT) assay. The combination index value was utilized to analyze the degree of drug interaction, while trypan blue assay was applied to analyze the cells proliferation effect. The mineralization and detection of bone BSP and Osx genes were determined via histochemical staining and PCR test, respectively. Results: The EC_50_ of osteoblasts treated with TA and a 75:25 ratio of TA and PAM were more potent with lower EC_50_ at 0.56 µg/mL and 0.48 µg/mL, respectively. The combination of TA and PAM (75:25) was shown to have synergistic interaction. On Day 7, both TA and PAM groups showed significantly increased proliferation compared with control and combination groups. On Day 7, both the TA and combination-treated groups demonstrated a higher production of calcium deposits than the control and PAM-treated groups. Moreover, on Day 7, the combination-treated group showed a significantly higher expression of BSP and Osx genes than both the TA and PAM groups. Conclusion: Combination treatment of TA and PAM at 75:25 ameliorated the highest enhancement of osteoblast proliferation and mineralization as well as caused a high expression of BSP and Osx genes.

## 1. Introduction

Bone is a dynamic tissue that constantly undergoes two processes throughout life, namely, modeling and remodeling, to grow or change shape. Bone modeling is a process by which bones change shape or size in response to physiologic influences or mechanical forces that are encountered by the skeleton, whereas bone remodeling takes place so that the bone may maintain its strength and mineral homeostasis [1]. Bone remodeling is tightly regulated by a cross-talk between bone-forming osteoblasts and bone-resorbing osteoclasts [2]. Osteoblasts, which are bone-forming cells, secrete collagenous and non-collagenous proteins, including osteocalcin, osteopontin, osteonectin, bone sialoprotein (BSP), and bone morphogenetic proteins (BMPs) [3]. In vitro data suggest that BSP may initiate hydroxyapatite crystal formation in the bone matrix [4]. In addition, an active osteoblast secretes the earliest bone formation marker, which is osterix (Osx). This will reduce the number of osteoblasts by converting it to osteocytes [5].

The disturbance in the bone remodeling process can lead to skeletal system disorder, such as osteoporosis. Osteoporosis is a metabolic bone disease caused by an imbalance between bone resorption and bone formation, where the rate of bone resorption is greater compared with bone formation [6]. Pamidronate (PAM) is a type of bisphosphonate that is mainly used in the treatment of osteoporosis by inhibiting bone resorption via osteoclasts during bone remodeling [7]. Apart from their inhibitory effect toward osteoclasts, PAM also enhances the production of collagen type I synthesized by osteoblasts. Osteoblast proliferation and extracellular matrix synthesis, particularly collagen type I, may have an anabolic effect on bone. The enhanced bone density mediated by bisphosphonates appears to be caused by the stimulation of osteoblast differentiation [8]. However, bisphosphonate is poorly absorbed and needs to be taken separately from food [9] while demonstrating some adverse effects [10]. In that regard, the present community has shifted their interest to natural products, such as herbal medicines [11].

Polyphenols are examples of phytochemicals that are widely found in plants, which have many medicinal values, such as protection against the development of cancer, cardiovascular diseases, diabetes, neurodegenerative diseases, and osteoporosis [12]. Polyphenols have antioxidant properties, which can prevent osteoporosis by scavenging reactive oxygen species (ROS), down-regulating inflammatory mediators, and help in bone formation by up-regulating bone formation markers, such as runt-related transcription factor-2 (Runx2), osteocalcin, Wnt signaling pathway, β-catenin, and insulin-like growth factor (IGF)-1 [13,14]. Tannic acid (TA) is a polyphenol classified from tannin group with antioxidant properties [15,16]. Antioxidants activate the differentiation of osteoblasts as well as prevent the action of ROS directly [17]. The usage of TA in the permissible limits is able to benefit mankind, as it possesses numerous medicinal benefits. Nevertheless, it was reported that a high-dose consumption of TA either through ingestion or inhalation may induce moderate toxicity such as vomiting, nausea, constipation, abdominal pain, and liver damage [18]. Thus, extensive investigation on the effect of TA is necessary to ensure the safety as well as to maximize its medicinal benefit.

In 2012, Ko et al. [19] investigated the combination effects of a Chinese herbal medicine called *Herba epimedii*, *Ligustri lucidi fructus*, and *Psoraleae fructus* (ELP) with anti-resorptive medications (alendronate and raloxifene) in rats to prevent osteoporosis. The herbal formula has been shown to promote osteogenic differentiation in rat mesenchymal stem cells by elevating alkaline phosphatase (ALP) activity and matrix calcium deposition. Drug combination studies usually aim to achieve synergistic treatment effect and toxicity mitigation as well as to minimize or delay the induction of drug resistance [20]. The net effects of drug combination include synergism or additive effect, antagonism or subtractive effect, and alteration of the effect of one or more drugs [21]. 

In the previous study, the combination of PAM and semi-purified fractions of *Quercus infectoria* (SFQI) (containing gallic acid, digallate, ellagic acid, phaseolic acid, syringic acid, and theogallin) showed positive effects on the proliferation and differentiation of osteoblasts. The levels of bone formation markers (Runx2, BMP-2, and Osx) were higher in the combination treatment group compared with those of groups treated with each agent [22,23]. Moreover, the authors found that the treatment with synthetic TA enhances human fetal osteoblast cell line (hFOB 1.19) proliferation, causes morphology changes, and improves mineralization [24]. Hence, the aim of this study is to evaluate the effect of polyphenol TA alone or in combination with PAM on proliferation, synergistic interaction, mineralization, as well as the expression of BSP and Osx genes in hFOB 1.19 cells.

## 2. Results

### 2.1. EC_50_ of TA and Combination of TA with PAM on hFOB 1.19 Cells

**Figure 1:**
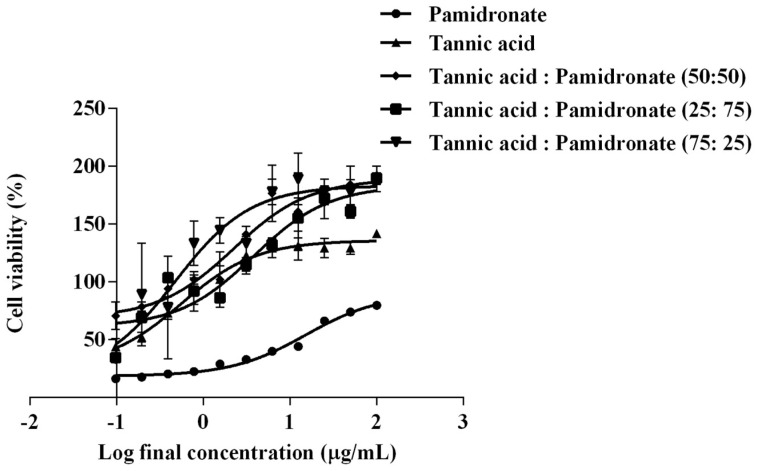
Percentage of cell viability (%) against log concentration of tannic acid (TA) alone, pam–dronate (PAM) alone, and percentage combination at the ratio of TA:PAM at 50:50, 25:75, and 75:25. The viability of hFOB 1.19 cells was determined by MTT assay. The values were expressed in mean ± SEM from three independent (*n* = 3) experiments.

MTT assay was done to investigate the effect of TA and different combinations of TA and PAM toward the hFOB 1.19 cell line compared to PAM as positive control. The cell treated with DMSO only served as negative control. The MTT assay illustrates that the TA and combinations of TA with PAM stimulated better cellular viability compared with PAM alone as the control drug. Figure 1 shows the percentage viability of hFOB 1.19 cells against log concentration of the treatment and control used. The EC_50_ for TA was observed to be lower (0.56 µg/mL) than that of the control drug of PAM (15.27 µg/mL). In the combination-treated group, the combination of TA with PAM at percentage ratios at 50:50, 25:75, and 75:25 showed EC_50_ values at 2.25 µg/mL, 3.80 µg/mL, and 0.48 µg/mL, respectively (Table 1). The combination of TA and PAM at a 75:25 ratio showed the lowest EC_50_ compared with the other two combination treatments. The EC_50_ of TA, PAM, and a combination of TA with PAM at a ratio of 75:25 were henceforth used to treat the cells in subsequent experiments.

### 2.2. Synergistic Activity of TA and PAM

The combination effect of TA and PAM on hFOB 1.19 cells was determined by using Compusyn software (ComboSyn, Inc., Paramus, NJ, USA). Table 2 showed the combination index (CI) value for the combination of TA and PAM at each percentage ratio. Synergistic activity was determined when TA and PAM were combined at the percentage ratio of 75:25 with the CI value obtained shown below 1, which is at 0.48. Meanwhile, the combination of TA and PAM at percentage ratios of 50:50 and 25:75 showed a CI value of 2.25 and 3.80, respectively. A CI higher than 1 indicates the antagonistic interaction between TA and PAM.

### 2.3. Proliferative Activity of hFOB 1.19 Cells

**Figure 2:**
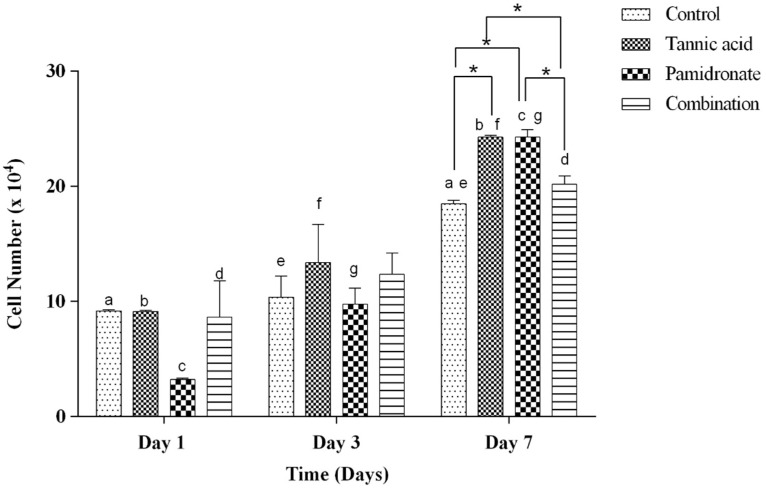
The number of live cells (×10^4^) at Day 1, 3, and 7 in the control group (DMSO treated), tannic acid (TA)-treated group, pamidronate (PAM)-treated group, and the combination of TA and PAM (75:25) determined by Trypan blue exclusion assay. All data are shown as the mean (SEM) of three independent experiments. Days that share the same letter display a significant difference within the same group (*p* < 0.05), whereas * shows a significant difference among the four groups in similar days (*p* < 0.05) by one-way ANOVA and Tukey’s test.

The number of viable hFOB 1.19 cells in response to the treatment with TA, PAM, and a combination of TA with PAM at the percentage ratio of 75:25 were in a time-dependent manner. Figure 2 shows the hFOB 1.19 cell numbers in response to the treatment for Day 1, Day 3, and Day 7. All groups in Day 7 showed significant differences when compared to Day 1 and Day 3, except for the combination group, which only showed significant differences when comparing between Day 1 and Day 7. On Day 7, both the treated groups of TA alone and PAM alone showed a significant increase in cell proliferation rate than that of the control (untreated) and combination groups.

### 2.4. Formation of Mineralized Calcium and Phosphate Deposits

**Figure 3:**
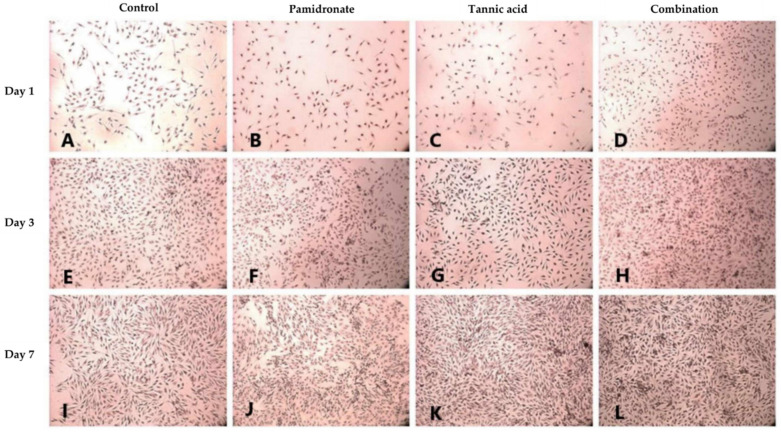
Alizarin red S (ARS) staining is used to evaluate calcium-rich deposits by cells in culture (**A**–**L**). ARS staining for calcium (Ca) deposits in hFOB 1.19 cells for 1, 3, and 7 days in the control group (DMSO treated), tannic acid (TA)-treated group, pamidronate (PAM)-treated group, and the combination of TA and PAM (75:25) observed using an image analyzer (magnification: 22×). ARS reveals the presence of Ca in mineralizing cells by forming a red–orange spots calcium complex.

**Figure 4:**
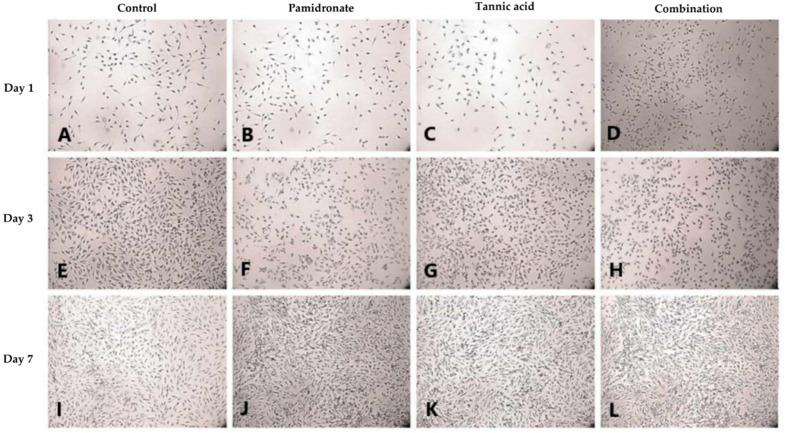
In cell culture, von Kossa staining using silver nitrate is widely used to detect mineralization (**A**–**L**). von Kossa staining for phosphate (P) deposits in hFOB 1.19 cells for 1, 3, and 7 days in the control group (DMSO treated), tannic acid (TA)-treated group, pamidronate (PAM)-treated group, and the combination of TA and PAM (75:25) observed using an image analyzer (magnification: 22×). The reaction between silver nitrate in von Kossa and P formed black spots, which represents phosphate deposits.

Alizarin Red S (ARS) staining was done to identify the presence of calcium deposits in the matrix by observing the bright red appearance of the cell under an image analyzer. On Day 1, and Day 3, the combination treatment of TA and PAM stimulated more calcium deposition on the samples than the other three groups (Figure 3A–H). On Day 3, the TA-treated group appeared to produce the least calcium deposition. However, on Day 7, both the TA and combination-treated groups had more calcium deposits than the untreated and PAM-treated groups (Figure 3I–L). Additionally, von Kossa staining was used to confirm the presence of phosphate-containing matrix, which illustrated to be in dark brown when viewed through an image analyzer. As demonstrated in Figure 4A–L, the combination-treated group produced more phosphate on Day 1 due to the presence of more black stains under the microscope. Nevertheless, on Day 3, both the untreated and TA-treated groups produced more phosphate. Meanwhile, on Day 7, all three treated groups produced more phosphate than the untreated group (Figure 4I–L).

### 2.5. Expression of BSP and Osx Genes

**Figure 5:**
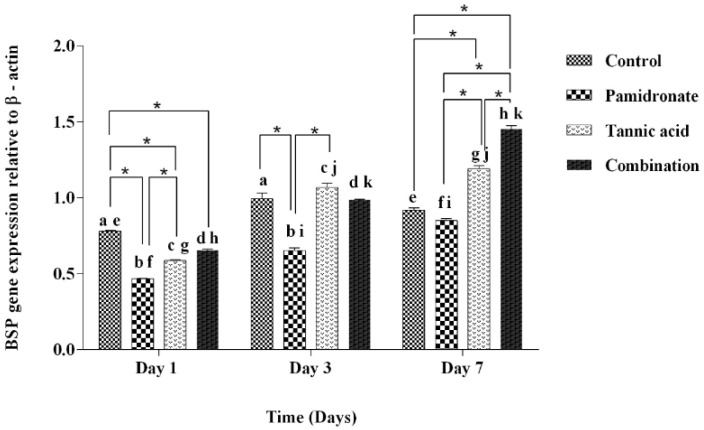
The gene expression of BSP relative to β-actin at Days 1, 3, and 7 in hFOB 1.19 cells expressed by control (DMSO treated), pamidronate (PAM), tannic acid (TA), and combination treatment (TA + PAM) groups analyzed by PCR. All data are shown as the mean (SEM) of three independent experiments. Days that share a similar letter display a significant difference within the same group (*p* < 0.05), whereas * shows a significant difference among the four groups in similar day (*p* < 0.05) by one-way ANOVA and Tukey’s test.

**Figure 6:**
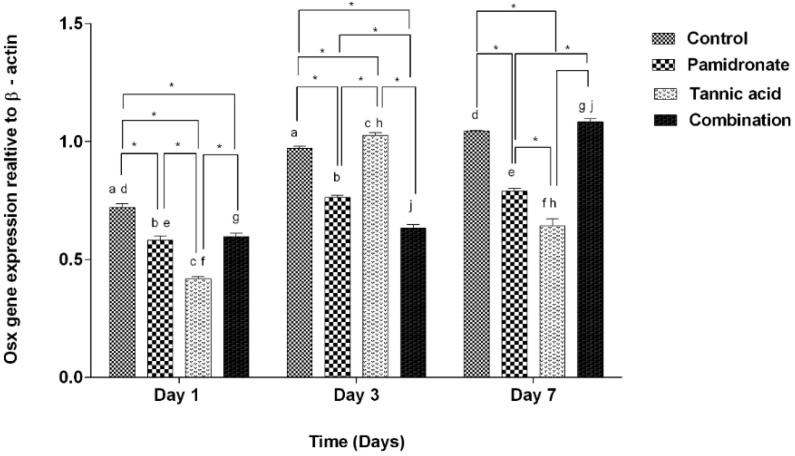
The gene expression of Osx relative to β-actin at Days 1, 3, and 7 in hFOB 1.19 cells expressed by control (DMSO treated), pamidronate (PAM), tannic acid (TA), and combination treatment (TA + PAM) groups, analyzed by PCR. All data are shown as the mean (SEM) of three independent experiments. Days that share the similar letter display a significant difference within the same group (*p* < 0.05), whereas * shows significant difference among the four groups in similar days (*p* < 0.05) by one-way ANOVA and Tukey’s test.

To strengthen the findings of this work, the authors of this research also investigate the gene expression of BSP and Osx by PCR. Figure 5 demonstrates that BSP was upregulated in hFOB1.19 cells treated with TA, PAM, and a combination of TA:PAM in a time-dependent manner prior to treatment at Day 1, Day 3, and Day 7 except for the control (untreated) group. On Day 7, the combination-treated group demonstrated significantly higher expression of BSP genes than the other three groups (TA, PAM, and control groups) (Figure 5). However, on Day 1, the BSP was downregulated to 0.60-fold and 0.75-fold for both PAM and TA groups, respectively (*p* < 0.001), while it was upregulated to 1.26-fold for the combination treatment group (*p* < 0.001) when compared to the control group. While, on Day 3, compared to the control group, there was a downregulation of BSP for PAM and the combination treatment group with 0.66-fold (*p* < 0.001) for both groups. There was slight increment for the TA group compared to the control group by 1.44-fold (*p* = 0.211). On Day 7, only the PAM-treated group showed a decrease in BSP expression by 0.92-fold (*p* = 0.111), while both TA and the combination-treated group showed enhanced BSP expression by 1.30-fold (*p* < 0.001) and 1.58-fold (*p* < 0.001), respectively by comparing to the control group. In the combination-treated group, the results of relative Osx gene expression are almost aligned with the BSP gene expression. On Day 7, the combination-treated group demonstrated a significantly higher expression of Osx genes than both TA and PAM-treated groups. Meanwhile, for the TA-treated group, there was a significant reduction in the expression of Osx on Day 7 compared with Day 3 (Figure 6). By comparing to the control group, on Day 1, there was a decrease in Osx expression in all treatment groups, with PAM showing a decrease by 0.81-fold (*p* = 0.001), TA by 0.58-fold (*p* < 0.001), and combination group by 0.93-fold (*p* = 0.002). On Day 3, only the TA-treated group showed a significant inclined of Osx expression by 1.05-fold (*p* = 0.035), while both PAM and the combination group showed a decline in Osx expression by 0.80-fold (*p* < 0.001) and 0.68-fold (*p* < 0.001), respectively. Concurrently, on Day 7, only the combination-treated group showed an upregulation of the Osx gene by 1.06-fold (*p* = 0.425); in contrast, the PAM and TA-treated groups showed downregulation by 0.76-fold (*p* < 0.001) and 0.67-fold (*p* < 0.001), respectively. In addition, Figure 5 and Figure 6 indicate that the hFOB 1.19 cells treated with a combination treatment of TA and PAM expressed the highest level of BSP and Osx at Day 7 of treatment. The current study suggested that the combination of TA and PAM is a more effective anabolic agent for promoting bone metabolism in hFOB 1.19 cells via upregulation of the osteoclastogenic markers BSP and Osx.

## 3. Discussion

Tannic acid (TA) is a secondary metabolite from plants, which belongs to the polyphenol family, with a chemical formula of C_76_H_52_O_46_, and it is composed of a central glucose unit and hydroxyl groups that have been completely esterified by phenols—a structure that is similar to other polyphenols [25]. It can be found in green tea, coffee, red wine, nuts, and most plants [26]. Various studies suggest that polyphenols can be used to treat osteoporosis by increasing the proliferation of osteoblasts due to their antioxidant properties [27]. In this study, it was shown that TA was a more effective treatment with an EC_50_ of 0.56 µg/mL compared with PAM, which had an EC_50_ of 15.27 µg/mL. This finding shows that the concentration of TA needed to promote and enhance the proliferative activities of hFOB 1.19 cells is lower compared with that of PAM. Garcia-Martnez et al. [28] observed that phenolic extracts from extra virgin olive oil promote osteoblast proliferation in vitro, which is consistent with our finding. An in vivo study done by Tomaszewska et al. [29] found that a diet high in TA increased bone volume in heavy metal-poisoned rats by decreasing trabecular separation and increasing trabecular thickness. Meanwhile, bisphosphonates can increase osteoblast proliferation at low doses but decrease it at high doses [30]. 

To enhance treatment efficacy while minimizing the adverse effects of PAM, this research performed evaluation on the combined effect of TA and PAM at three different percentage ratios of 50:50, 75:25, and 25:75. In comparison with the other two ratios, the combination of TA and PAM at a ratio of 75:25 had the lowest EC_50_ (0.48 µg/mL). The pharmacological interactions between the three combination ratios were evaluated using the CompuSyn software program by calculating the combination index (CI) value. Based on the findings, a combination at a percentage ratio of 75:25 exhibited a synergistic effect (CI of 0.6372), whereas the other two ratios exhibited an antagonistic effect (CI more than 1). In the application of combination therapy, synergistic effects were the most desired drug–drug interactions [31]. For example, the combination of estrogen with bisphosphonate for the treatment of postmenopausal is often given to the patient rather than a single agent, as it is more effective in increasing bone mineral density (BMD) [32]. Thus, in subsequent experiments, the cells were treated with TA and PAM in a 75:25 ratio, which not only increased treatment efficacy but also reduced the side effects of PAM.

Upon undergoing any treatment, hFOB 1.19 cells were examined for matrix calcium and phosphate deposits. Calcium and phosphate deposits were chosen for observation because calcium phosphate (CaP) is necessary for the formation of CaP-containing vesicles, also called matrix vesicles, during bone mineralization [33]. In addition, the presence of calcium ions (Ca^2+^) stimulates osteoblast cell differentiation and proliferation [34,35]. Mineral deposition per viable cell increased in a time-dependent manner in cells treated with TA, PAM, or a combination of TA and PAM. The authors of this research found that CaP both resided primarily intracellularly in mineralizing cells. From the ARS and von Kossa stainings, both the TA and combination-treated groups consistently produced more CaP deposits. Increased ARS and von Kossa staining intensities indicate increased mineral content [36,37]. Later, the precipitation of CaP followed by oxidation resulted in the formation of hydroxyapatite on crosslinked collagen fibers, which had led to bone formation [38]. Tian et al. [39] reported that the addition of TA to hydroxyapatite composites resulted in increased osteogenesis and angiogenesis, as indicated by immunohistochemistry staining for osteocalcin (OCN) and vascular endothelial growth factor (VEGF). Thus, treatment with TA alone or in combination with PAM appears to be more effective than the control and PAM-treated groups at increasing osteoblastic mineralization 

The reverse transcriptase polymerase chain reaction (RT-PCR) was used in this study, with ribonucleic acid (RNA) as the starting template. In comparison to DNA, RNA is used because messenger RNA (mRNA) is rapidly degraded in viable cells, with the majority of mRNA species having half-lives measured in minutes. Therefore, it is believed that using RT-PCR to detect mRNA is a more accurate indicator of cell viability [40]. The beta (β)-actin gene was used as a housekeeping gene because it encodes a structural protein of the cytoskeleton that is present in all cells [41]. The human body contains both enzymatic and non-enzymatic antioxidant systems, which include metal chelating proteins and endogenous antioxidant enzymes, such as catalase, glutathione peroxidase, and superoxide dismutase. Antioxidants act as radical scavengers [42]. Macronutrients and micronutrients, collectively referred to as natural antioxidants, can enter cells and interact with transcription factors, thereby activating target gene expression. Certain polyphenols, such as epigallocatechin gallate (EGCG), resveratrol, and icariin, can activate osteoblast-related genes by regulating a transcription factor. After 48 h of treatment, EGCG (a green tea catechin) significantly increased the expression of osterix (Osx) in murine bone marrow mesenchymal stem cells [43,44]. Osx plays an important role in osteoblast differentiation due to its ability to regulate bone sialoprotein (BSP) expression [45]. 

BSP is a glycosylated, highly sulfated, and phosphorylated protein that is expressed in mineralizing tissue. BSP is primarily produced by mature osteoblasts, and its expression is positively correlated with bone production [46]. Conversely, Osx is a zinc-finger transcription factor that is a member of the specificity protein (Sp) family. It is required for the differentiation of pre-osteoblast to mature osteoblast, as Osx gene expression increases with osteoblast differentiation [47,48,49]. In this study, BSP and Osx genes were expressed throughout the entire treatment periods (Day 1, Day 3, and Day 7), demonstrating that mineralization, a characteristic of osteoblast proliferation and differentiation, had formed in response to TA, PAM, and combination treatments. Both BSP and Osx are involved in the wingless-int (Wnt) pathway by participating in the differentiation of mesenchymal cells into osteoblast progenitors [50]. Moreover, Osx has long been recognized as a critical transcription factor for osteoblast differentiation and bone mineralization, suggesting that it may play a role in the development of novel therapeutic strategies for bone diseases [51].

On Day 7, the results of this study demonstrate that BSP and Osx gene expression levels are significantly higher in the combination-treated group than in the TA or PAM alone groups. The current findings corroborate our previous biochemical findings that hFOB 1.19 cells treated with a combination of semi-purified fractions isolated from Quercus infectoria and PAM expressed the highest levels of bone formation markers (BMP-2 and Runx2) on Day 7 [22]. In addition, from Day 3 to Day 7, expression of the Osx gene increased significantly in the combination-treated group, whereas it decreased significantly in the TA-treated group. It is believed that the synergistic effect of the combination of TA and PAM might play a prominent role in boosting the expression of both genes.

The current study reported that the combination of TA and PAM had a synergistic effect in osteoblasts by upregulating the expression of BSP and Osx genes. However, many specific and non-specific bone formation markers, such as bone alkaline phosphatase (BALP), osteocalcin (OCN), runt-related transcription factor 2 (Runx2), bone morphogenetic-2 (BMP-2), procollagen Type I N-terminal propeptide (PINP), procollagen Type I C-terminal propeptide (PICP), and osteoprotegerin (OPG), are not yet included in this study. Incorporating a variety of bone formation markers at the gene and protein levels could elucidate the molecular mechanism underlying the effect of TA and PAM in osteoblasts. Moreover, Scanning Electron Microscopy-Energy X-ray (SEM-EDX) can be utilized to determine the qualitative assessment of calcium and phosphate deposits in osteoblasts. Hence, it is crucial to further investigate the expression of the related bone formation markers that play a vital role in the regulation of osteoblast differentiation as well as bone mineralization to validate the current findings.

## 4. Materials and Methods

### 4.1. Cell Culture

#### 4.1.1. Cell Revival and Subculture

Human fetal osteoblast cell line hFOB 1.19 (CRL-11372) was purchased from American Type Culture Collection (ATCC^®^) (Manassas, VA 20110). The hFOB 1.19 cells were cultured in Dulbecco’s Modified Eagle Medium F-12 nutrient mixture (DMEM/F12) (Invitrogen GmBH, Karlsruhe, Germany) supplemented with 10% fetal bovine serum (FBS) (Invitrogen GmBH, Karlsruhe, Germany) and 1% penicillin/streptomycin serum (Invitrogen GmBH, Karlsruhe, Germany) [22,52]. The cells were incubated at 37 °C in a humidified atmosphere of 95% air and 5% CO_2_ (Sheldon, Cornelius, OR, USA) to provide the optimum temperature, moisture (sterile environment), and pH. The cells were monitored closely for 24 h. All cell culture-related works were conducted in Biosafety Cabinet (BSC) Class II to maintain sterility conditions.

#### 4.1.2. Preparation for Cell Treatment

Tannic acid (TA) (Chemfaces, Daejeon, Korea) and pamidronate (PAM) (Toronto Research Chemical, North York, ON, Canada) were prepared by diluting in dimethyl sulfoxide (DMSO) (Nacalai Tesque, Kyoto, Japan). The TA used is a naturally extracted compound from fruit and seeds of *Phyllanthus emblica* (Indian gooseberry). In this experiment, PAM-treated cells acted as a positive control, while cells treated with DMSO only served as a negative control. The treatment groups comprised cells treated with TA and a combination between TA and PAM that were combined at several percentage ratios (v:v), which are 75:25, 50:50, and 25:75. TA and PAM were prepared at a concentration of 10 mg/mL. Both TA and PAM were combined at this concentration according to the percentage ratios selected. Then, all of the prepared solutions were serially diluted before treating the cells to give the final concentration at 99, 49.5, 24.8, 12.4, 6.19, 3.09, 1.55, 0.77, 0.39, 0.19, and 0.01 µg/mL.

Cells were initially seeded at 5 × 10^4^ cells/mL in 96-well plates and incubated overnight at 37 °C in 5% CO_2_ incubator for cellular adherence. Then, the cells were treated with serially diluted agents that were previously prepared. The treated cells were incubated in a 5% CO_2_ incubator at 37 °C for 24 h (Day 1), 72 h (Day 3), and 168 h (Day 7), respectively. The tests were conducted in three independent experiments in triplicate to ensure the reliability and acceptance of the results obtained.

### 4.2. MTT Assay

The 3-(4,5-dimethyl-2-thiazolyl)-2,5-diphenyl-2H-tetrazolium bromide (MTT) assay (Nacalai Tesque, Kyoto, Japan) was performed to determine cell viability. The protocol was modified from Hassan et al. [53]. Cultured cell with more than 80% confluency was used; in this test, yellow MTT was reduced to purple formazan. A stock solution of 5 mg/mL MTT dye (Nacalai Tesque, Japan) was prepared using Dulbecco’s phosphate-buffered saline (Thermo Fisher Scientific, Waltham, MA, USA). Then, 15 µL of freshly prepared MTT dye was added to each well, and the plate was incubated for 4 h [54]. After the removal of the supernatant, 100 μL of DMSO was added to all wells to solubilize the formazan crystals formed, and the plate was further incubated for 15 min. The optical density (OD) was measured at 570 nm by using the ELISA microplate reader. The percentage of the viable cell was calculated by using the following formula: % viable cells = (OD value of treated cells/OD value of untreated cells) × 100. The dose–response curve of cell viability (%) against the final concentration was plotted, and the half maximal effective concentration (EC_50_) value was identified using GraphPad Prism software version 7 (GraphPad Software, San Diego, CA, USA).

### 4.3. Analysis of Synergistic Activity

TA and PAM at the initial concentration of 10 mg/mL were combined according to the selected ratios (75:25, 50:50, 25:75). Then, the combined agents were serially diluted to produce concentrations of 10, 5, 2.5, 1.25, 0.625, 0.313, 0.156, 0.078, 0.039, 0.02, and 0.01 mg/mL. The percentage viabilities of the cells in response to the treatment with each serially diluted combination agent were determined and used for combination analysis. Synergistic interaction between TA and PAM was determined via the combination index (CI) method that was described by Chou [55]. The CI allows for the quantitation of multiple drug interactions based on the calculation using the CI equation. The CI value was measured automatically with the CompuSyn program (ComboSyn Inc., Paramus, NJ, USA). The CI value obtained specifies the degree of drug interactions, in which CI <1 indicates a synergistic effect between the two drugs used: CI = 1, which indicates an additive effect, and CI >1, which indicates antagonistic effects.

### 4.4. Trypan Blue Exclusion Assay

Proliferation assay was conducted to determine the number of hFOB 1.19 cells treated with TA, PAM, and combination of TA:PAM at Days 1, 3, and 7 via the utilization of trypan blue exclusion assay. The cells were plated at 5 × 10^3^ cells/well with 100 µL culture medium per well in a 96-well microtiter plate and left overnight prior to attachment. TA, PAM, and a combination of TA:PAM were added into each well at different concentrations, and the cells were incubated at 5% CO_2_ in a 37 °C humidified incubator. The cells were trypsinized with 100 µL trypsin/EDTA and incubated for 3 min for the cells to detach. Subsequently, the cells were viewed by using an inverted microscope to confirm that 90% detachment had occurred. To stop the trypsinization process, 200 µL of culture medium was added, and the cells were resuspended. Then, the cells were stained with trypan blue exclusion dye solution for cell counting. Automated cell counting was carried out using Countess^TM^ Automated Cell Counter (Invitrogen, Waltham, MA, USA). The graph number of viable cells versus time of each treatment and control group was plotted and analyzed using the IBM SPSS Statistics 24.

### 4.5. Histochemical Assays for Mineralized Calcium and Phosphate Deposits

The formation of mineralized calcium and phosphate in the cells was determined by Alizarin Red S staining for calcium deposition and von Kossa staining for phosphate deposition and at Days 1, 3, and 7.

#### 4.5.1. Alizarin Red S Staining

The staining protocol was slightly modified from Gregory et al. [56]. The fixed cells were stained with 40 mM Alizarin Red (pH 4.1 to 4.3; 1 mL/well) (Sigma-Aldrich, St. Louis, MO, USA) and were incubated for 20 min at room temperature. Alizarin Red S forms complexes with calcium ions. Excess dye was removed by washing the samples four (4) times with distilled water (dH_2_O) until the rinsed solution was clear. Calcium deposits within cell layers appeared as spots and were stained red-orange. The representative images were acquired using an image analyzer (Olympus, Tokyo, Japan).

#### 4.5.2. Von Kossa Staining

The staining protocol was slightly modified from Brauer et al. [57]. The fixed cells were stained with 5% silver nitrate (AgNO_3_) solution (1 mL/well) for 30 min under ultraviolet light. The silver ions in the reagent reacted with phosphate present in the cells. After removing the AgNO_3_ solution, the cell layers were washed two (2) to three (3) times with dH_2_O, and 1 mL of 5% sodium thiosulfate was added to remove excess silver salts. The cell layers were washed 2 to 3 times with distilled water for 3 to 5 min. Phosphate deposits within the cell layers appeared as spots and were stained black. The representative images were acquired using an image analyzer (Olympus, Tokyo, Japan).

### 4.6. Gene Expression by Polymerase Chain Reaction (PCR)

RNA was isolated using commercially available kit (Total RNA Mini Kit, Geneaid). The integrity and purity of the total RNA were verified using NanoDrop Reader. Isolated RNA was reverse-transcribed with a Tetro cDNA synthesis kit (Bioline, London, UK) according to the manufacturer’s protocol. Each reverse-transcription reaction contained 1 µg RNA. The amplification kit used in this research was MyTaq^TM^ Mix (Bioline, UK). The housekeeping gene, β-actin, was used as endogenous control to normalize calculation by using the comparative CT method. Table 3 shows the primer sequence used in this study. The reaction mixture was subjected to 95 °C for 1 min, which was followed by 35 cycles at 95 °C for 15 s (denaturation); 60 °C for 15 s (annealing); and lastly, 72 °C for 10 s (extension). For agarose gel electrophoresis, 10 µL of each amplified product was analyzed by electrophoresis on 2% agarose gel in 1X Tris-acetate-EDTA (TAE) buffer at 90 volts for 45 min. Two µL of SYBR Safe DNA gel stain (Thermo Fisher Scientific, USA) was added to gel prior to solidifying, and 2 µL of 100 bp DNA ladder was used as a DNA size standard. Data analysis was carried out using the J Image software (LOCI, University of Wisconsin, Madison, WI, USA) by comparing target gene bands to β-actin band before running ANOVA using Statistical Package of Social Sciences (SPSS) Software, version 24 (IBM, Armonk, NY, USA).

### 4.7. Statistical Analysis

The data obtained were expressed in mean ± SEM (standard error mean) from three independent experiments (*n* = 3). For the half-maximal effective concentration (EC_50_), data were analyzed by using GraphPad Prism software, version 7 for the determination of the EC_50_ value. For the proliferation assay, the data obtained were initially tested for the normality and homogeneity of variance through the Shapiro–Wilk test. Next, statistical comparison was conducted via the utilization of one-way ANOVA with Tukey’s Honest Significant Difference (HSD) post hoc test. The result was considered statistically significant if *p* < 0.05. Each analysis was conducted using the Statistical Package of Social Sciences (SPSS) software, version 24.

## 5. Conclusions

In this current study, it is observed that TA alone and a combination treatment of TA and PAM had the potential to promote cell proliferation, thus enhancing the mineralization of the matrix by increasing the level of calcium and phosphate depositions as well as the expression of BSP and Osx genes. Hence, our data demonstrated that the combination of TA with PAM has high potential to be developed as one of the therapeutic regimens for osteoporosis treatment with minimal adverse effects to human.

## Figures and Tables

**Table 1:** Value of half maximal effective concentration (EC_50_) for treatment and control group on hFOB 1.19 cells.

Treatment	TA	PAM	50:50 (TA:PAM)	25:75 (TA:PAM)	75:25 (TA:PAM)
EC_50_ (µg/mL)	0.56	15.27	2.25	3.80	0.48
Log_10_ EC_50_	−0.25	1.18	0.35	0.58	−0.32

**Table 2:** Combination index (CI) value for the combination of tannic acid (TA) and pamidronate (PAM).

Combination Ratio (TA:PAM)	Combination Index (CI)	Indication
50:50	2.0826	Antagonism
25:75	1.8831	Antagonism
75:25	0.6372	Synergism

**Table 3:** Primer sequence used in quantitative PCR analysis.

Gene	Sense (5′-3′)	Antisense (5′-3′)
Bone sialoprotein (BSP)	AATGAAAACGAAGAAAGCGAAG	ATCATAGCCATCGTAGCCTTGT
Osterix (Osx)	TGCGAAGCCTTGCCATACA	TCCTCCTGCGACTGCCCTAA
β-actin	GGCATCGTGATGGACTCCG	GCTGGAAGGTGGACAGCGA

## Data Availability

Not applicable.
